# Immunoproteomics Reveals Pathogen’s Antigens Involved in *Homo sapiens*–*Histoplasma capsulatum* Interaction and Specific Linear B-Cell Epitopes in Histoplasmosis

**DOI:** 10.3389/fcimb.2020.591121

**Published:** 2020-10-29

**Authors:** Marcos Abreu Almeida, Rodrigo Almeida-Paes, Allan Jefferson Guimarães, Richard Hemmi Valente, Célia Maria de Almeida Soares, Rosely Maria Zancopé-Oliveira

**Affiliations:** ^1^ Laboratório de Micologia, Instituto Nacional de Infectologia Evandro Chagas, Fundação Oswaldo Cruz, Rio de Janeiro, Brazil; ^2^ Departamento de Microbiologia e Parasitologia, Instituto Biomédico, Universidade Federal Fluminense, Niterói, Brazil; ^3^ Laboratório de Toxinologia, Instituto Oswaldo Cruz, Fundação Oswaldo Cruz, Rio de Janeiro, Brazil; ^4^ Instituto de Ciências Biológicas, Universidade Federal de Goiás, Goiânia, Brazil

**Keywords:** *Histoplasma capsulatum*, *Homo sapiens*, immunoproteome, mass spectrometry, epitopes, M antigen, YPS-3, catalase P

## Abstract

Histoplasmosis is one of the most frequent systemic mycosis in HIV patients. In these patients, histoplasmosis has high rates of morbidity/mortality if diagnosis and treatment are delayed. Despite its relevance, there is a paucity of information concerning the interaction between *Histoplasma capsulatum* and the human host, especially regarding the B-cell response, which has a direct impact on the diagnosis. Culture-based “gold-standard” methods have limitations, making immunodiagnostic tests an attractive option for clinical decisions. Despite the continuous development of those tests, improving serological parameters is necessary to make these methods efficient tools for definitive diagnosis of histoplasmosis. This includes the determination of more specific and immunogenic antigens to improve specificity and sensitivity of assays. In this study, we performed a co-immunoprecipitation assay between a protein extract from the yeast form of *H. capsulatum* and pooled sera from patients with proven histoplasmosis, followed by shotgun mass spectrometry identification of antigenic targets. Sera from patients with other pulmonary infections or from healthy individuals living in endemic areas of histoplasmosis were also assayed to determine potentially cross-reactive proteins. The primary structures of *H. capsulatum* immunoprecipitated proteins were evaluated using the DNAStar Protean 7.0 software. In parallel, the online epitope prediction server, BCPREDS, was used to complement the B-epitope prediction analysis. Our approach detected 132 reactive proteins to antibodies present in histoplasmosis patients’ sera. Among these antigens, 127 were recognized also by antibodies in heterologous patients’ and/or normal healthy donors’ sera. Therefore, the only three antigens specifically recognized by antibodies of histoplasmosis patients were mapped as potential antigenic targets: the M antigen, previously demonstrated in the diagnosis of histoplasmosis, and the catalase P and YPS-3 proteins, characterized as virulence factors of *H. capsulatum*, with antigenic properties still unclear. The other two proteins were fragments of the YPS-3 and M antigen. Overlapping results obtained from the two aforementioned bioinformatic tools, 16 regions from these three proteins are proposed as putative B-cell epitopes exclusive to *H. capsulatum*. These data reveal a new role for these proteins on *H. capsulatum* interactions with the immune system and indicate their possible use in new methods for the diagnosis of histoplasmosis.

## Introduction

Histoplasmosis is a life-threatening systemic mycosis with worldwide distribution, with predominance areas in the Americas (Wheat et al., 2016), caused by the dimorphic fungus *Histoplasma capsulatum* (Teixeira et al., 2016). The mycelial form of *H. capsulatum* is found in the environment and the yeast form is observed during parasitism. Under laboratory conditions, the mycelium form can be cultivated from 25 to 30°C; a temperature switch—up to between 35 and 37°C—in enriched media can reversibly induce the yeast morphotype in this fungus (Sahaza et al., 2020).

In general, infection starts upon inhalation of airborne infectious propagules of *H. capsulatum* that are highly resistant to adverse environmental factors (Frías-De-León et al., 2017). Microconidia are the most frequent infectious elements because of their small size, which facilitates penetration into the pulmonary alveoli. This event is followed by the conversion of the fungus into yeasts, which has been considered a critical factor for the pathogenicity of *H. capsulatum* (Knox and Hage, 2010).

The infection by *H. capsulatum* depends on a complex interaction between the fungus and the mammalian host, with disease prognosis determined by factors such as immune status of the host, strain virulence, and inhaled fungal burden (Sepúlveda et al., 2014). Although histoplasmosis affects either immunologically intact or deficient hosts, individuals with compromised cellular immune response presents more severe manifestations of this disease (Mittal et al., 2019). The yeast cells of *H. capsulatum* are highly adapted to the host since they can survive and reproduce within phagocytic cells (Garfoot and Rappleye, 2016). *Histoplasma capsulatum* strategies against macrophages include evasion from the immune response on entry, inactivation of nitrogen and oxygen reactive species, prevention of phagolysosomal fusion, hindrance of lysosomal pH reduction, siderophore production, and induction of apoptosis for escape and dissemination into the host (Long et al., 2003; Missall et al., 2004; Guimarães et al., 2011; Hilty et al., 2011; Mittal et al., 2019).

Although the cellular immune response is long recognized as relevant in the control of *H. capsulatum* infection, roles for antibodies in the pathogenesis of histoplasmosis have been proposed. In fact, a monoclonal antibody reactive against a cell surface histone-like protein has proved to be protective in a murine histoplasmosis model (Nosanchuk et al., 2003). Also, monoclonal antibodies against heat shock protein 60 (HSP60) can alter the fate of the fungus within phagocytic cells and change host cytokine production profiles (Guimarães et al., 2009), as well as modify qualitatively and quantitatively the gene expression and contents of extracellular vesicles secreted by yeast cells of the fungus, impacting on the effector functions of bone-marrow derived macrophages (Baltazar et al., 2018; Burnet et al., 2020). Moreover, antibodies against the M and H antigens, a catalase B and beta-glucosidase, respectively, are consistently produced by patients with different clinical forms of histoplasmosis and, therefore, are largely applied in serodiagnostic tests (Pizzini et al., 1999; Guimarães et al., 2004; Almeida et al., 2016; Almeida et al., 2019).

Routine serodiagnosis of histoplasmosis is usually performed with the crude supernatant of *H. capsulatum* mycelial cultures known as histoplasmin. This is composed predominantly by C, H, and M antigens. The C antigen is a galactomannan responsible for the cross-reactions observed with other fungal species. The H antigen is a β-glucosidase and the M antigen is a catalase. These last two proteins are widely used as targets for antibody detection in the diagnosis of histoplasmosis (Guimarães et al., 2006).

The use of proteomic methodologies can assist in the identification of proteins involved in the parasite-host interaction process (Pastorelli et al., 2006). Besides clarifying the pathogenesis of mycotic diseases, the recognition of immunologically reactive molecules can aid in the development of more efficient serological tests for diagnosis of infections and differentiation of close-related species (Moreira et al., 2020). Immunoproteomic approach has been successfully applied to identify specific antigens in infectious diseases, as demonstrated for several fungal pathogens such as *Aspergillus fumigatus* (Virginio et al., 2014), *Candida albicans* (Pitarch et al., 2016), *Coccidioides posadasii* (Tarcha et al., 2006), *Cryptococcus gattii* (Martins et al., 2013), *Cryptococcus neoformans* (Neuville et al., 2000), *Paracoccidioides* spp. (Moreira et al., 2020), and *Sporothrix schenckii* (Rodrigues et al., 2015).

In this work, an immunoproteomic approach was employed to characterize *H. capsulatum* proteins specifically recognized by antibodies exclusively present in histoplasmosis patient’s serum samples and, therefore with a role in the host-pathogen interaction. Moreover, these specific antigens were identified and their B-epitopes characterized. Consequently, their applications could improve the serodiagnosis of histoplasmosis.

## Materials and Methods

### Microorganism and Culture Conditions

*Histoplasma capsulatum* G-217B (ATCC 26032) was used in this study. This is a reference strain used by different research groups worldwide. It has been genotyped and classified in the NAm2 phylogenetic group, which has been proposed to be renamed as the cryptic species *Histoplasma ohiense* (Sepúlveda et al., 2017). The fungus was grown in Ham’s F-12 medium supplemented with glucose (18.2 g/L), glutamic acid (1 g/L), 4- (2-hydroxyethyl) -1-piperazinoethanesulfonic acid [HEPES (6 g/L)], and cysteine (8.4 mg/L), pH 7.5. Yeast cells were grown at 36°C for 72 h under constant agitation (150 rpm).

### Human Sera

The serum samples used were obtained from patients recruited at the Evandro Chagas National Institute of Infectious Diseases, a reference center for Infectious Diseases in Rio de Janeiro, Brazil. The study included samples from three groups: (i) 10 independent patients with histoplasmosis—confirmed by the isolation of *H. capsulatum* in culture—presenting different clinical manifestations [pulmonary (n = 3), mediastinal (n = 2), and disseminated (n = 5)], (ii) 10 independent patients with culture-proven diseases other than histoplasmosis [aspergillosis (n = 2), coccidioidomycosis (n = 2), cryptococcosis (n = 2), and paracoccidioidomycosis (n = 2)], as well as tuberculosis (n = 2), and (iii) Group NHS—10 samples from different healthy individuals were also included in this study. One serum sample was collected from each participant of the study. These serum samples were stored at −20°C until use. Three pools of sera were built with the samples included in this study and named as follows: (i) histoplasmosis (HPM), (ii) heterologous (HET), and (iii) normal healthy sera (NHS). The use of these samples was approved by the Research Ethics Committee of the Evandro Chagas National Institute of Infectious Diseases–Fiocruz, accession number CAAE 0029.0.009.000-11.

### Preparation of Protein Extract

Yeast cells were collected by centrifugation (10.000 g, 10 min, 4°C) and washed three times with phosphate buffered saline (PBS; 1.4 mM KH_2_PO_4_, 8 mM Na_2_HPO_4_, 140 mM NaCl, 2.7 mM KCl; pH 7.2). The protein extracts were obtained by mechanical maceration in the presence of liquid nitrogen until complete cell disruption, monitored by light microscopy. Next, extraction buffer (20mM Tris-HCl pH 8.8, 2mM CaCl_2_) supplemented with protease inhibitors (Complete Mini^®^, Roche Diagnostics, Manheim, Germany) and zirconia beads (0.5 mm) were added. The material was subjected to vigorous agitation in a Mini-Beadbeater (Biospec products, Bartlesville, OK, USA) for five 30 s cycles, intercalated with 1 min ice bath incubation. The lysate was then subjected to centrifugation at 10.000 g for 15 min at 4°C and the protein concentration (supernatant) was determined by the Bradford method, using bovine serum albumin as standard (Bradford, 1976). Samples were submitted to sodium dodecyl sulfate-polyacrylamide gel electrophoresis (1D SDS-PAGE) according to Laemmli (1970) and two-dimensional gel electrophoresis (2DE). Regarding 2DE, isoelectric focusing was carried out on 11-cm immobilized 3–10 pH gradient gel strips (Immobiline DryStrip gels, GE Healthcare Biosciences). Each protein extract (200 µg) was solubilized in 200 µl of rehydration solution [8 M urea, 2% (w/v) 3-[(3-cholamidopropyl)-dimethyl-ammonio]-1-propane sulfonate (CHAPS), 1% (w/v) ampholytes (IPG Buffer 3–10 linear), 0.1 M dithiotreitol (DTT), and 0.002% (w/v) bromophenol blue (BFB)]. The active rehydration and isoelectric focusing steps were performed in an Ettan IPGphor system (GE Healthcare Biosciences) under the following conditions: 30 V for 12 h, 200 V for 1 h, 500 V for 1 h, 1,000 V for 1 h, 3,500 V for 30 min, and 6,000 V for 6 h. For the second dimension, proteins were reduced with 0.1% (w/v) DTT followed by alkylation with 0.4% (w/v) iodoacetamide; both steps in equilibrium buffer [1.5 M Tris-HCl pH 8.8; 6 M urea, 30% (v/v) glycerol, 2% (w/v) SDS, and 0.002% (w/v) BFB] at constant agitation for 15 min each step. After this preparation, the strips were placed on the top of 12% polyacrylamide gels with SDS (Laemmli, 1970) and the system sealed with 0.5% (w/v) agarose in Tris-glycine buffer. The electrophoretic run was carried out at 15°C on a Protean II system (Bio-Rad) under the following conditions: 3 mA for 30 min and 25 mA until the end of the run. The proteins were colloidal Coomassie blue- or silver-stained.

### Serological Tests

All sera included in this study were individually tested through the Outcherlony double immunodiffusion test using histoplasmin as an antigen (Guimarães et al., 2006). In addition, a Western blot assay (Pizzini et al., 1999) was performed to check the individual reactivity of the 30 included serum samples, as well as the three pool of sera described previously, against the protein extract prepared from the yeast cells of G217B *H. capsulatum* strain. Moreover, to check whether inespecific reactions could occur between the secondary antibody (alkaline phosphatase-conjugated AffiniPure goat anti-human IgG, Fc fragment specific, Jackson ImmunoResearch, West Grove, PA, USA) and the protein extract, a conjugate control was performed, where only the secondary antibody was added to the nitrocellulose strip with the antigen.

### Co-Immunoprecipitation Assay

For the co-immunoprecipitation step, magnetic microspheres—Dynabeads^®^ Protein G (Invitrogen, California, USA)—were used, following a previously described protocol (Moura et al., 2011). In the antibody-binding step, 50 μl of the solution containing the microspheres were resuspended and separated on a magnetic platform for 5 min and the supernatant was removed. The microspheres were washed three times (5 min each) in 200 µl of PBS with 0.1% Tween 20 (PBS-T). Ten micrograms of protein from each serum pool (HPM, HET, and NHS) were individually analyzed. As an additional control, the microspheres were incubated with PBS only (CON). Experimental triplicates were performed for each condition. Each serum pool was diluted to 50 µg/ml of total proteins in PBS and 200 µl of the dilution was applied to the beads and incubated for 2 h at room temperature under rotation. Again, the microspheres were separated, and washed three times in PBS-T after supernatant disposal. For the crosslinking step, 200 µl of a 13 mg/ml DMP (dimethyl pimelimidate) solution in PBS (pH 9.0) were added and incubated for 15 min under rotation at room temperature. The microspheres were subjected to three washes in PBS-T, as described above. At the immunoprecipitation stage, 25 µg of *H. capsulatum* protein extract, prepared as described above, diluted in 200 µl of PBS-T were incubated with the magnetic microspheres and submitted to rotation at room temperature for 2 h. Again, microsphere separation and washing steps were carried out. For the elution step, the microspheres were resuspended in 100 µl of PBS-T and transferred to a new tube. The polypeptides were eluted in 50 µl of a solution containing 0.1 M glycine (pH 2.8) incubated for 5 min at room temperature under rotation. The microspheres were separated for 5 min, the supernatant removed, and the pH adjusted to 7.0. Subsequently, the samples were dried on a centrifugal vacuum concentrator (Speed-Vac, Thermo) and stored at −20°C until further processing.

### Shotgun Mass Spectrometry (MS) Protein Identification

For each dried sample resulting from the coimmunoprecipitation assays (above), 100 µg of protein were submitted to trypsin digestion. Samples were initially resuspended in 20 µl of a solution containing 0.4 M ammonium bicarbonate, 8 M urea, followed by addition of 5 µl of 0.1 M dithiotreitol and incubation at 37°C for 30 min. Then, 5 µl of 0.4 M iodoacetamide were added and incubated for 15 min at room temperature in the dark. Samples were diluted by addition of 130 µl of Milli-Q water followed by trypsin (Promega, Wisconsin, USA) addition at 1/50 (m/m) of enzyme to substrate; first incubation for 16 h at 37°C and second incubation at 56°C for 45 min; reaction was stopped with 20 µl of 10% (v/v) formic acid. Samples were then desalted with in-lab-generated columns packed with Poros R2 resin (Life Technologies). Columns were initially activated with 100% acetonitrile (CH_3_CN), followed by equilibration with 1% (v/v) trifluoroacetic acid (TFA). Samples were applied to the columns and subjected to five washes with 0.1% TFA solution. The elutions were carried out with four washes of 0.1% TFA in 70% CH_3_CN. Samples were dried on a centrifugal vacuum concentrator (SpeedVac, Thermo) and stored at −20°C until use. Prior to MS, each sample was resuspended in 20 µl of 1% formic acid. MS analysis was conducted in technical triplicate (4 µl injection per replicate) for samples HPM, HET, and NHS and technical duplicate for sample CON. Briefly, peptides were submitted to reversed-phase nanochromatography (EASY-nLC II, Thermo) coupled to a high-resolution nano-electrospray ionization mass spectrometer (LTQ Orbitrap XL, Thermo), using the data-dependent analysis mode with MS1 spectra being acquired in the orbitrap analyzer and MS2 spectra in the linear trap. Detailed experimental settings were the same as previously described (Brunoro et al., 2015), to the exception of column length, gradient duration, and number of most intense ions submitted to CID for each spectra, which were 20 cm, 168 min, and 7 ions/spectra, respectively, in the present work.

### Data Analysis

All MS/MS spectra were analyzed using PEAKS Studio 8.5 (Bioinformatics Solutions, Canada). After data refinement with the precursor (mass only) correction option, PEAKS DE NOVO analysis was run assuming trypsin digestion, with a fragment ion mass tolerance of 0.60 Da and a parent ion tolerance of 10 ppm. Cysteine carbamidomethylation (+57.02 Da) was set as fixed modification and methionine oxidation (+15.99 Da) as variable modification; a maximum of three variable modifications per peptide was allowed. PEAKS DB analysis was performed using these same parameters plus the possibility of up to two missed enzyme cleavages and non-specific cleavage at one side of the peptides. Searches were made against an NCBInr database subset for “*H. capsulatum*” (37,754 entries). False discovery rates (FDR) were estimated through the PEAKS decoy fusion approach, and only peptide/protein identifications with FDR values ≤1% were accepted. Identified proteins were subjected to *in silico* analysis using UniProt database (www.uniprot.org) and WoLF PSORT server (https://wolfpsort.hgc.jp/). UniProt database was used to categorize the proteins according to their function and WoLF PSORT server to predict subcellular localization of the proteins. Presence of the proteins within *H. capsulatum* extracellular vesicles (EVs) was performed comparing our proteomic data with data previously described on proteomics of EVs (Albuquerque et al., 2008; Baltazar et al., 2018). After that Venn diagrams were created using the Venny 2.1 tool (http://bioinfogp.cnb.csic.es/tools/venny/) to determine which proteins were recognized by the serum pool of patients with histoplasmosis (HPM) detected in the three experimental replicates. From this intercession, new Venn diagrams were constructed with all proteins recognized by the serum pool of patients with other infections (HET), serum pool of normal healthy sera (NHS), and with all proteins recognized in the control experiment. In the HET, NHS, and control conditions, proteins identified in at least one of the replicates were considered in the exclusion analysis, in order to determine which proteins were specifically recognized by antibodies present in serum samples from patients with histoplasmosis. Proteome data could be found on: 10.17605/OSF.IO/FVMGE.

### *In Silico* Protein Similarity and Antigenicity Analysis

The polypeptide sequences of proteins identified from coimmunoprecipitation, and which reacted specifically with the HPM serum pool, were submitted to the basic local alignment search tool for proteins (BLASTp) to search for sequence similarity. For antigenicity analysis, the amino acid sequences were evaluated using the software DNAStar Protean 7.0 (DNASTAR Inc.), by the Jameson-Wolf algorithm, associated with the hydrophobicity index (Kyte-Doolittle) and the surface accessibility probability of the protein (Emini). In parallel, in order to complement the B epitope prediction, the amino acid sequences were analyzed by the epitope prediction online server, BCPREDS (http://ailab.ist.psu.edu/bcpred/index.html). The primary sequences of the proteins were given as input and the prediction of B cell epitopes was restricted to epitopes of fixed length of 20 amino acids. The standard specificity of 75% was used, and for the output of the results, the option of presenting only non-overlapping epitopes. The sequences of antigenic proteins and its possible epitopes demonstrated by analysis in bioinformatics tools had their homology analyzed against proteins from other pathogenic organisms that could have a confounding factor with histoplasmosis, using BLASTp.

## Results

### Profile of Proteic Extract

**Figure 1:**
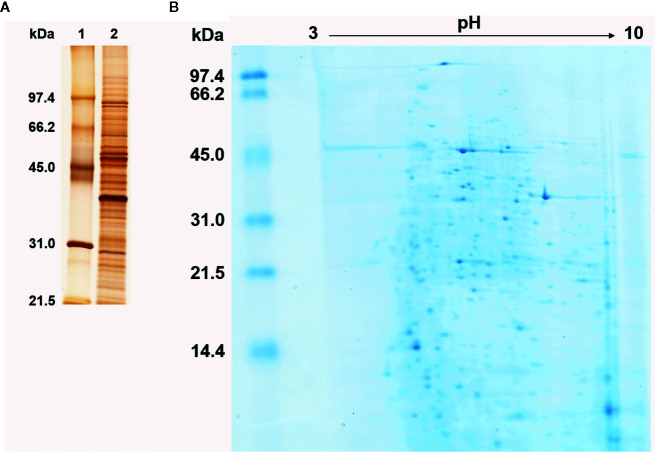
Protein profile of *Histoplasma capsulatum* yeast cell extract. **(A)** Silver stained SDS-PAGE under reducing conditions. 1, Molecular mass standard; 2, Yeast cell extract of *H. capsulatum*. Numbers on the left correspond to the molecular mass (kDa) of the standards. **(B)** Colloidal Coomassie blue stained 2DE. The linear pH gradient range is indicated above the gel and the molecular mass standards (kDa) are on the left.

*Histoplasma capsulatum* yeast protein extract was analyzed by SDS-PAGE and 2DE. Even though no quantitative evaluation was made, the extract displayed a heterogeneous proteic migration profile (as expected), with a broad distribution of molecular masses (**Figure 1A**, lane 2 and **Figure 1B**, vertical axis) and most proteins focusing in the *ca.* 5 to 8 pI range, although several proteins displayed high pI (>8) migration; this was not the case for the acidic region (pI < 5) (**Figure 1B**, horizontal axis).

### Immunoreactivity of Individual Serum Samples

All but one serum sample (HPM6) from patients with histoplasmosis were reactive in the double immunodiffusion test against histoplasmin, while all heterologous and normal human sera were negative in this regular serodiagnostic test. When the reactivity of these sera was tested against the protein extract produced in this study a diverse profile of antigenic recognition was observed among the individual sera, with patients with histoplasmosis presenting more recognized antigens than healthy individuals, as expected. Patients with paracoccidioidomycosis and aspergilosis also presented reactivity against a large number of antigenic proteins, while the reactivity was lower in sera from patients with cryptococcosis, coccidioidomycosis, and tuberculosis (**Supplementary Figure 1**).

### Immunoproteomics

**Figure 2:**
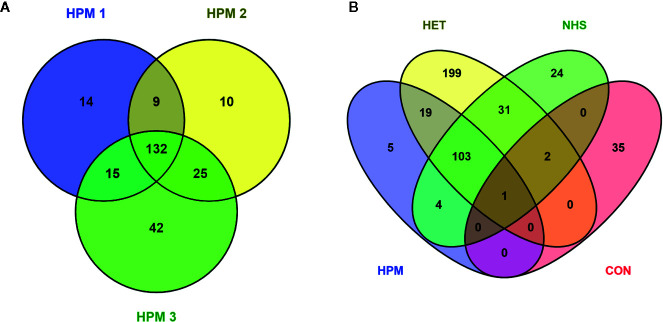
Venn diagrams depicting *Histoplasma capsulatum* proteins (potential antigens) recognized by the different pooled sera groups. **(A)** Number of proteins recognized by the pooled sera of patients with histoplasmosis (HPM) for the three biological replicates analyzed (HPM 1, HPM 2, and HPM 3); **(B)** The 132 proteins in common to all conditions in panel A were compared to the total list of proteins detected in at least one of the biological replicates for the other conditions: HET, pooled sera of patients with other fungal infections or tuberculosis; NHS, pooled sera of normal healthy donors; CON, mock control: phosphate buffered saline.

Proteomic analysis of potential antigen recognition by antibodies present in the pooled sera of patients with different clinical forms of histoplasmosis (HPM) identified a total 247 proteins (**Table S1**). However, only 132 antigenic proteins were commonly detected among the three biological replicates analyzed, were selected for further studies (**Figure 2A** and **Table S5**). Assays with heterologous pooled sera of patients with other mycoses or tuberculosis (HET), pooled normal healthy donors sera (NHS), and mock PBS control (CON) identified 355 (**Table S2**), 165 (**Table S3**), and 38 (**Table S4**) proteins, respectively.

**Figure 3:**
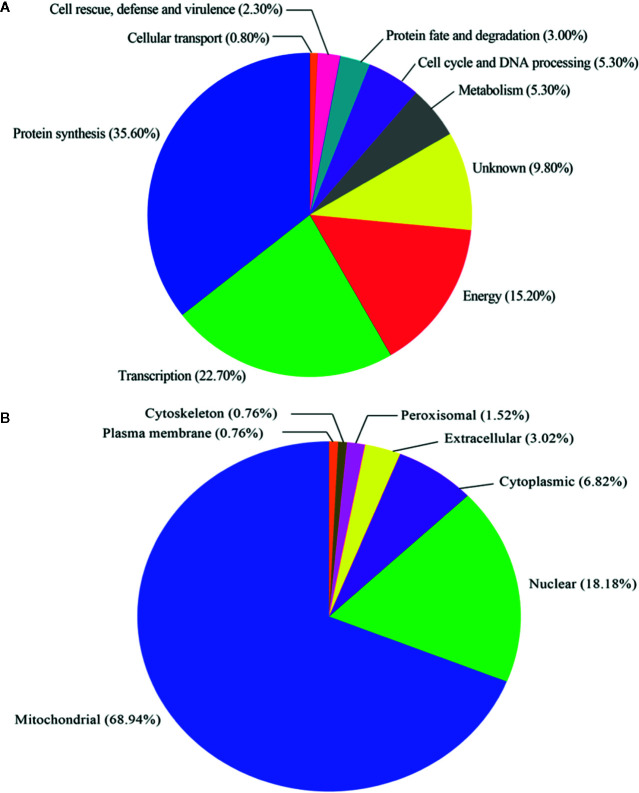
Functional classification and subcellular localization of immunoreactive proteins identified in *Histoplasma capsulatum*. **(A)** Functional classification of proteins based on UniProt database; **(B)** Subcellular localization of proteins based on the WoLF PSORT server.

The 132 proteins recognized in all HPM immunoproteomics data (**Table S5**) were classified according to the functional categories and subcellular localization, based on the UniProt database and WoLF PSORT server, respectively (**Figure 3** and **Table S6**). **Figure 3A** shows that most proteins were related to protein synthesis (47; 35.60%), followed by transcription (30; 22.70%), energy (20; 15.20%), metabolism (7; 5.30%), cell cycle and DNA processing (7; 5.30%), protein fate (4; 3.00%), cell rescue, defense, and virulence (3; 2.30%), and cellular transport (1; 0.80%). Thirteen proteins with unknown function totalized 9.80%. The prediction of subcellular localization of proteins revealed that most of them were classified as mitochondrial (91; 68.94%), followed by nuclear (24; 18.18%), cytoplasmic (9; 6.82%), extracellular (4; 3.02%), peroxisomal (2; 1.52%), plasma membrane (1; 0.76%), and cytoskeleton (1; 0.76%), as shown in **Figure 3B**.

Among the 132 antigenic proteins recognized by antibodies present in sera from patients with histoplasmosis, five protein entries were specifically recognized by these antibodies, without cross-reaction with antibodies present in sera from other conditions (**Figure 2B** and **Table S5**), namely: catalase (C0NVF6), catalase B (Q9Y7C2), M antigen (O13373), Yeast phase specific protein (Q8J1T0), and YPS-3 protein (Q00950). A BLASTp analysis of the identified catalase (C0NVF6) revealed 100% identity and query cover with the catalase isozyme P of *H. capsulatum* (accession number EEH04495), and therefore we refer to this protein as catalase P from now on. When the sequences from these five protein entries were compared, using the BLASTp, results showed that two of these entries consisted of fragments from larger proteins, which were also identified as specific through the strategy herein employed. Thus, after the removal of duplications, there were only three proteins supposedly with antigenic activity and specifically recognized by antibodies in sera of histoplasmosis patients, with high applicability on the diagnosis of histoplasmosis: catalase P, M antigen, and YPS-3.

### B-Cell Epitope Prediction of Specific Antigens of *Histoplasma capsulatum*

**Figure 4:**
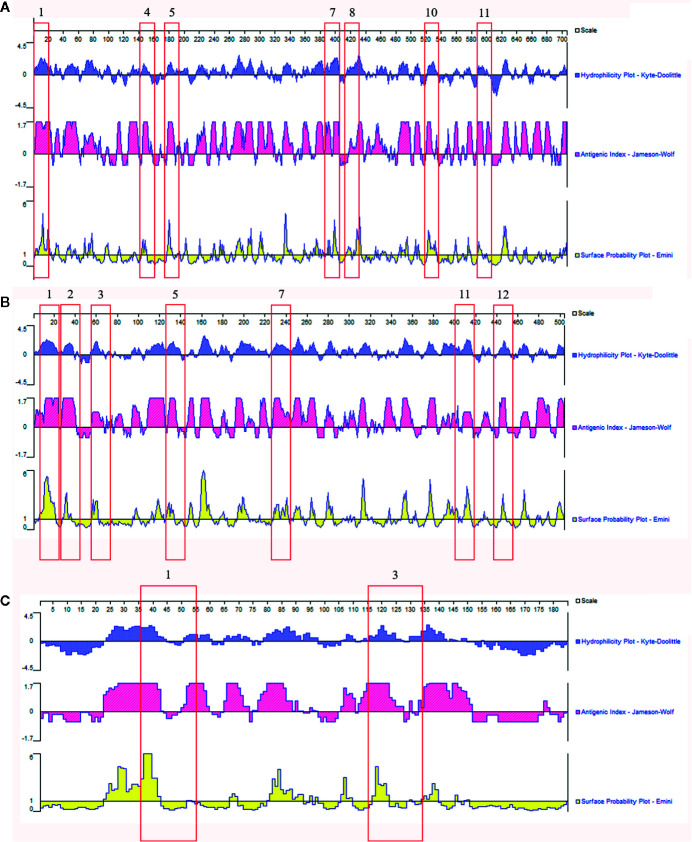
*In silico* evaluation of putative B epitopes on candidate proteins for antigenic targets for the diagnosis of histoplasmosis. Results obtained from overlapping data from two bioinformatics tools: the software DNAStar Protean 7.0 and BCPREDS server. **(A)** M antigen; **(B)** Catalase P; **(C)** YPS-3. The flagged numbers refer to the peptides described in **Table S7**.

The three specific candidate proteins as antigenic targets were then analyzed by the DNAStar software, combining the determination of antigenicity indexes (Jameson-Wolf algorithm), the hydrophobicity index (Kyte-Doolitle algorithm), and the probability of accessibility to the surface of the protein (Emini algorithm). As an additional analysis, an epitope prediction software (BCPREDS) was used, and 13 regions were proposed as possible B epitopes within the M antigen, 12 regions in the catalase P, and only 3 regions in the YPS-3 protein (**Table S7**). For identification of possible specific *H. capsulatum* B cell epitopes, the results obtained in the two bioinformatics tools (DNAStar and BCPREDS) were overlapped. Taking into account regions with high antigenic indices, combined with the hydrophobicity index and the probability of localization on the surface of the molecule, and considering only B cell epitopes having a BCPREDS score >0.8, sixteen regions have been proposed as the most immunogenic. Seven regions in M antigen, seven in catalase P, and two in YPS-3 protein, as shown in **Figure 4**. Finally, for further evaluation of the specificity of these epitopes, we performed an *in silico* analysis of their homology with proteins from other pathogens causing fungal diseases or infectious diseases that can be misdiagnosed as histoplasmosis. These data are presented in **Table S8**. In brief, it was observed a degree of similarity among epitopes previously showed in *Blastomyces dermatitidis* and *Tallaromyces marneffei*. However, four epitopes, two from catalase P (epitopes 1 and 12), one from the M antigen (epitope 1), and one from YPS-3 (epitope 1) presented low sequence identity with the organisms tested and could be considered specific for *H. capsulatum* (**Table S8**).

## Discussion

Histoplasmosis is a systemic mycosis much more widespread than the literature and epidemiological reports demonstrate (Antinori, 2014). Although a high endemicity was demonstrated in the Americas, the current knowledge of its distribution is incomplete (Bahr et al., 2015). Despite the relevance of this disease, there is a paucity of information regarding the interaction between *H. capsulatum* and the human host, especially regarding the B-cell response, which has a direct impact on the diagnosis of the infection.

The gold standard diagnosis of histoplasmosis occurs by demonstration of the yeast-like *H. capsulatum* cells on microscopic examination and isolation of the fungus in culture of clinical specimens. However, these methods may require invasive medical procedures, the culture test is time-consuming, and conversion to the yeast form is essential, requiring up to 8 weeks to reveal fungal growth. Thus, in many situations, the diagnosis of histoplasmosis is based on serological tests to detect antibodies and/or antigens (Almeida et al., 2019). On the other hand, much remains to be done to improve the laboratory diagnosis. The serological tests available still have limitations (Azar and Hage, 2017), and the discovery of new antigenic targets would be essential to improve the diagnosis of histoplasmosis and possible development of vaccines, since there is still no vaccine available against *H. capsulatum* (Roth et al., 2019).

The use of proteomic tools for the identification of proteins involved in the parasite-host interaction, and presumed to be antigenic targets, has been applied to pathogenic fungi studies (Crockett et al., 2012; Ball et al., 2019; Moreira et al., 2020). Thus, our work used co-immunoprecipitation followed by mass spectrometry to identify proteins involved the interplay between host and pathogen, specifically those recognized by B-cell receptor and their B-cell antibodies products, as with the identification of presumed antigenic targets with possible application in diagnostic tests.

A total of 132 antigenic proteins were recognized by antibodies from patients with histoplasmosis. Most of these molecules were ribosomal proteins related to protein synthesis and transcription. However, proteins with relevant roles in the parasite-host interaction have also been identified within this pooled. The list included proteins that contribute to the survival of *H. capsulatum* after oxidative stress produced by the innate immune system, such as the alternative oxidase and catalases B and P, may be important for virulence of this fungus (Johnson et al., 2003; Holbrook et al., 2013). Also, a cell surface antigen of *H. capsulatum*, histone 2B, related with binding to host cells and that has the ability to modulate the intracellular fate of the fungus (Nosanchuk et al., 2012), was identified. Another find was YPS-3 protein, localized in the cell wall and culture supernatants of *H. capsulatum*, which may influence the dimorphic transition. The *yps-3* gene expression is not fundamental for the transformation to the yeast phase, but it may facilitate adaptive processes that allow mycelium-to-yeast transition and survival at elevated host temperatures. Moreover, YPS-3 has already been associated with increased fungal burden in phagocyte-rich tissues (Keath et al., 1989; Weaver et al., 1996; Bohse and Woods, 2007).

Other proteins identified in this study with reactivity to the sera of patients with histoplasmosis have already had their functions described in other models. The nascent polypeptide-associated complex (α, β_1_ and β_3_ subunits) was found in *Saccharomyces cerevisiae*, in which the deletion of one subunit causes downregulation of the remaining subunits at physiological growth temperature. Cells lacking both β -subunits are have not been able to grow at 37°C (Reimann et al., 1999). Mills and colleagues (2016) proposed that increased mitochondrial oxidation of succinate *via* succinate dehydrogenase and an elevation of mitochondrial membrane potential combine to drive mitochondrial reactive oxygen species production. Thus, the same could happen in *H. capsulatum*, since succinate dehydrogenase was reactive to sera from patients with histoplasmosis in this study. However, *H. capsulatum* produces catalases that break down H_2_O_2_ into H_2_O and O_2_, working as a protection against oxidative mechanisms of the host (Maresca and Kobayashi, 2000).

The dihydrolipoamide succinyltransferase protein has already been described as immunogenic and potentially protective in some bacteria (Nguyen et al., 1999; Zygmunt et al., 2001; Gilmore et al., 2003). In our study dihydrolipoamide succinyltransferase also showed antigenicity, even though not specific to *H. capsulatum*. This protein was also recognized by heterologous pooled sera and the normal healthy pooled sera.

It was surprising that most immunoreactive *H. capsulatum* proteins were predicted with intracellular localization. In fact, *in silico* analysis regarding the cellular location of these proteins revealed that most of them were present in mitochondria. However, we found literature data (Albuquerque et al., 2008; Baltazar et al., 2018) supporting that approximately 57% of the proteins found in our study have already been identified by proteomic tools when analyzing macromolecules secreted within extracellular vesicles of *H. capsulatum* (**Tables S6**). These proteins could, through EVs transport, be accessed by the immune system and activate B-cells.

Since the contents of the *H. capsulatum* EVs produced during parasitism are not fully characterized and such contents change, depending on the microenvironment (Baltazar et al., 2018; Cleare et al., 2020), it is possible that the intracellular proteins detected as immunogenic in our proteomic approach become available to the immune system through EVs produced in the mammalian host. Future studies should be performed to test such hypothesis.

Surprisingly, beta-glucosidase, also known as H antigen, was not identified in our proteomic approach. This molecule is reported as a secreted protein of mycelium and yeast cells of *H. capsulatum*, and high levels of the H antigen were detected in supernatants of G217B yeast cell cultures. However, antibodies anti-H are found in less than 20% of patients with histoplasmosis (Azar and Hage, 2017). This fact could explain the absence of this protein in our immunoproteomic assay.

Although a high number of *H. capsulatum* proteins are recognized by antibodies present in pooled sera from patients with histoplasmosis, most of them are also recognized by antibodies from patients with other infectious diseases or healthy individuals, indicating a certain degree of similarity on the immunopathogenesis of these diseases. Three proteins are proposed as specific antigenic targets: M antigen, catalase P, and YPS-3.

In previous studies, the M antigen had its biological nature identified, was structurally characterized and its expression was detected on the yeast cell surface; in addition, it was characterized as a useful exoantigen in the diagnosis of histoplasmosis (Zancopé-Oliveira et al., 1999; Guimarães et al., 2008). However, catalase P and YPS-3 protein have been studied and characterized as virulence factors in *H. capsulatum* (Johnson et al., 2002; Bohse and Woods, 2007), but not yet described as antigenic targets.

In *H. capsulatum*, catalase P has already been described as a virulence factor and is involved in the response to oxidative stress. It is referred to as a protein with similarity to the well-known peroxisomal catalases of animals and yeasts of the order *Saccharomycotina* (Johnson et al., 2002). The transcriptional expression of the *H. capsulatum* CATB (M antigen) and CATP (catalase P) genes was assessed by Northern blot and the results at the transcript levels are shown for three conditions: cell morphology, carbon source, and response to oxidative stress. These results demonstrated regulation of CATB and CATP only by the availability of different carbon sources (Johnson et al., 2002).

*Histoplasma capsulatum* was grown in different concentrations of H_2_O_2_ to evaluate the role of catalases in oxidative stress *in vitro*, presenting progressive reduction in growth with increased concentrations of hydrogen peroxide from 1.0 mM up to 1.6 mM. However, no growth occurred by 120 h in concentrations higher than 1.6 mM (Guimarães et al., 2008).

The YPS-3 protein, present in the cell wall and also released in culture medium, is produced only by the yeast phase of *H. capsulatum* (Bohse and Woods, 2005). Its location suggests that YPS3 is a protein capable of being recognized by antibodies and the results of the present study show for the first time the antigenic potential of this protein. A previous study proposed YPS3 to be virulence factor involved in the progression of the disseminated disease, since, when its production was blocked by interference RNA in animal infection, the mutants showed a significant decrease in fungal dissemination (Bohse and Woods, 2007).

Our data suggest that the three proteins identified as antigenic targets could be used in the development of alternative methods of histoplasmosis diagnosis. However, when assessing homology with other infectious agents, some B cell epitopes of M antigen and catalase P showed high rates of sequence identity with regions of *Blastomyces dermatitidis* and *Talaromyces marneffei*, reaching up to 100% identity (such as epitope 2 of catalase P, and epitopes 5, 8 and 10 of M antigen). This could be a problem in regions where there is an overlap of endemic areas of these diseases. However, we could not demonstrate this situation since serum samples from patients with blastomycosis, frequent in the Mississippi and Ohio River valleys (US) and Midwestern states and Canadian provinces that border the Great Lakes, and areas adjacent to the Saint Lawrence Seaway (Castillo et al., 2016), and talaromycosis endemic to Southeast Asia (Chan et al., 2016) are not available in our country. Consequently, they were not included in the heterologous pooled sera being a limitation of this study. In order to circumvent this limitation, avoiding possible cross reactions, more specific B cell epitopes could be used as targets in possible immunoassays, such as epitopes 1 and 12 of catalase P, and epitope 1 of M antigen, which showed low identity (values between 42 and 78% of identity) with regions of *B. dermatitidis* and *T. marneffei*. Future studies regarding the application of YPS-3 and the aforementioned epitopes of M antigen and catalase P are necessary to improve the immunodiagnosis of histoplasmosis.

Another relevant limitation of the study is that only one *H. capsulatum* strain was evaluated. This strain is the holotype of the proposed *H. ohiense* cryptic species and one of the most studied *Histoplasma* strains around the world. It is possible that different proteins are involved in the host-pathogen interaction of other phylogenetic clades. Future studies with different clades and cryptic species are necessary to a deeper knowledge of the immunopathogenesis of histoplasmosis.

The major aim in this study was to detect specific antigenic proteins that could be used to develop future assays to be employed in endemic areas of histoplasmosis. Therefore, the use of a control group from an endemic area would improve the detection of such proteins. It is expected that individuals living in non-endemic areas of histoplasmosis have less cross-reacting antibodies against *H. capsulatum*, and therefore, the three proteins identified as specific in this study (M antigen, catalase P, and YSP-3) should be also suitable as diagnostic markers in non-endemic areas of this mycosis.

## Conclusion

The diagnosis and management of histoplasmosis remains a major public health problem in several countries. The knowledge the interplay between host and pathogen during infection and the ability to definitively diagnose histoplasmosis has become even more important due to the increasing number of patients susceptible to this disease. Proteomic tools could elucidate the mechanisms of fungal pathogenesis, the relationship between host and pathogen during infection, and also identify of antigenic targets.

This is the first study to use immunoproteomic approaches to identify proteins with a high antigenic index and map specific epitopes for the diagnosis of histoplasmosis. Besides that, bioinformatic tools made it possible to carry out other analyzes, such as functional classification and subcellular localization.

Three antigenic proteins of *H. capsulatum* are described in this study as putative candidates for the immunodiagnostic of histoplasmosis: catalase P, M antigen, and YPS-3. Also, 16 epitopes proposed as exclusive of *H. capsulatum* were mapped using bioinformatics. Among these, four epitopes are proposed as the most promising candidate once it shows lowest homology with proteins of other pathogens that could be misdiagnosed with histoplasmosis.

In conclusion, the three immunogenic proteins described in this work may be applied to histoplasmosis immunodiagnosis, as well as patient follow-ups, treatment, and putative vaccine candidate.

## Data Availability Statement

The original contributions presented in the study are included in the article/**Supplementary Material**. Further inquiries can be directed to the corresponding author.

## Ethics Statement

The studies involving human participants were reviewed and approved by the Research Ethics Committee of the Evandro Chagas National Institute of Infectious Diseases–Fiocruz, accession number CAAE 0029.0.009.000-11. Written informed consent for participation was not required for this study in accordance with the national legislation and the institutional requirements.

## Author Contributions

MAA, RA-P, and AJG performed the experiments. MAA, RA-P, AJG, and RHV analyzed and interpreted the data. CMAS and RMZ-O participated in the design of the study and revised this manuscript. All authors contributed to the article and approved the submitted version.

## Funding

This work was supported by grants from Conselho Nacional de Desenvolvimento Científico e Tecnológico (CNPq), Fundação de Amparo à Pesquisa do Estado do Rio de Janeiro (FAPERJ), and Fundação de Amparo à Pesquisa do Estado de Goiás (FAPEG). RA-P was supported in part by CNPq (305487/2015-9). AG was supported by CNPq (311470/2018-1) and FAPERJ (E-26/202.696/2018). RV is a fellow from CNPq (Grant 304523/2019-4). CS was supported by FAPEG/INCT (10267000022). RZ-O was supported in part by CNPq (302796/2017-7) and FAPERJ (E-26/202.527/2019). We are grateful for support from the Coordination for the Improvement of Higher Education Personnel (CAPES).

## Conflict of Interest

The authors declare that the research was conducted in the absence of any commercial or financial relationships that could be construed as a potential conflict of interest.

## Supplementary Material

Supplementary Figure 1 Immunologic reactivity of individual serum samples against the yeast protein extract of *H. capsulatum* G217B. Serum samples of patients with histoplasmosis (HPM1 to HPM10), paracoccidioidomycosis (HET1 and HET2), aspergilosis (HET3 and HET4), cryptococcosis (HET5 and HET6), coccidoiodomycosis (HET7 and HET8), and tuberculosis (HET9 and HET10), as well as serum samples from healthy individuals (NHS1 to NHS10) were tested through a Western blot assay against the protein extract used in this study. The reactivity of the three pool of sera used in the coimmunoprecipitation assay (HPM*, HET*, and NHS*) was also tested. MM, molecular mass standard (BioRad Laboratories Inc, Hercules, CA, USA); CC, conjugate control.
