# Supplementary figures and images for: Immunoproteomics Reveals Pathogen’s Antigens Involved in Homo sapiens–Histoplasma capsulatum Interaction and Specific Linear B-Cell Epitopes in Histoplasmosis
Source: Front Cell Infect Microbiol. 2020 Oct 29;10:591121. doi: 10.3389/fcimb.2020.591121 (PMC7673445; doi:10.3389/fcimb.2020.591121)

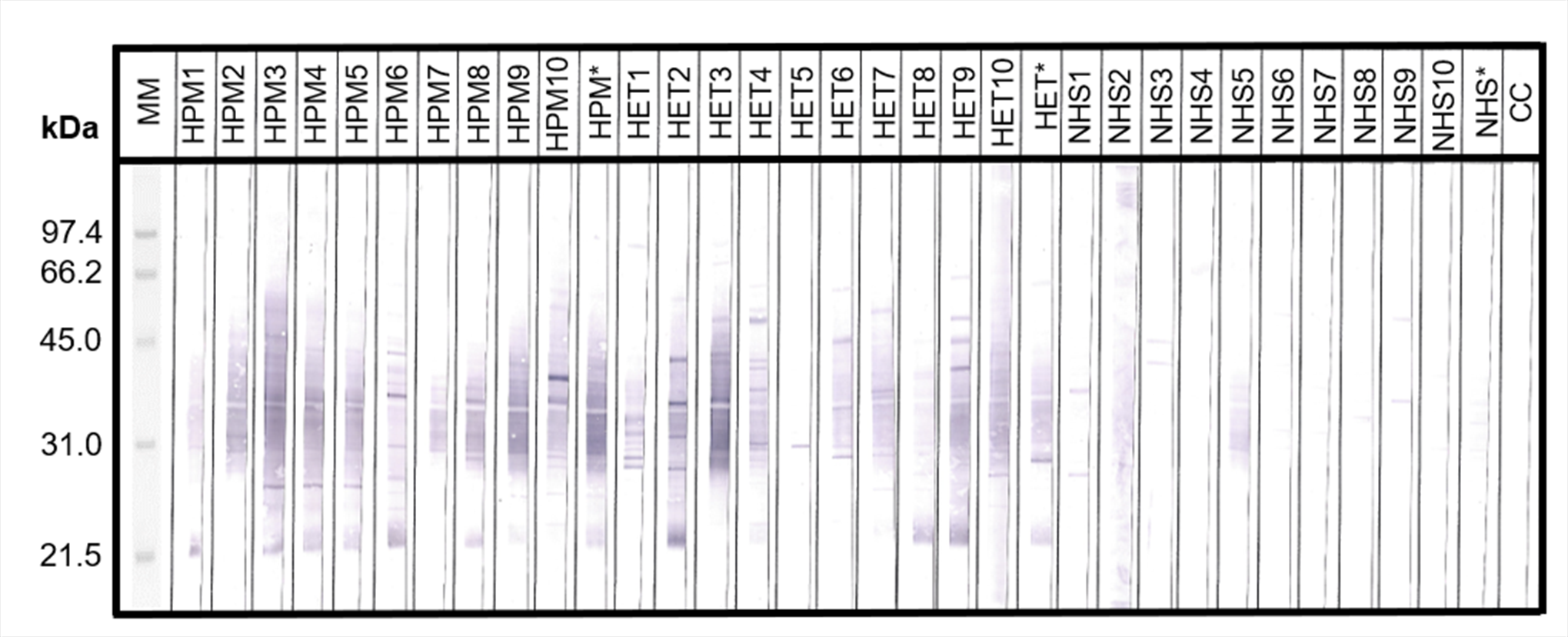

Supplement: Supplementary Figure 1 — Immunologic reactivity of individual serum samples against the yeast protein extract of H. capsulatum G217B. Serum samples of patients with histoplasmosis (HPM1 to HPM10), paracoccidioidomycosis (HET1 and HET2), aspergilosis (HET3 and HET4), cryptococcosis (HET5 and HET6), coccidoiodomycosis (HET7 and HET8), and tuberculosis (HET9 and HET10), as well as serum samples from healthy individuals (NHS1 to NHS10) were tested through a Western blot assay against the protein extract used in this study. The reactivity of the three pool of sera used in the coimmunoprecipitation assay (HPM*, HET*, and NHS*) was also tested. MM, molecular mass standard (BioRad Laboratories Inc, Hercules, CA, USA); CC, conjugate control. [file Image_1.tif]
